# Study on stability differences in heel kick movements of Tai Chi athletes based on statistical parametric mapping

**DOI:** 10.3389/fphys.2025.1629653

**Published:** 2025-08-29

**Authors:** Xiaopei Zhang, Gan Liu, Mengyao Jia, Yong Ma

**Affiliations:** ^1^ Engineering Research Center of Sports Health Intelligent Equipment of Hubei Province, Wuhan Sports University, Wuhan, China; ^2^ Key Laboratory of Sports Engineering of General Administration of Sports of China, Wuhan Sports University, Wuhan, China; ^3^ Research Center of Sports Equipment Engineering Technology of Hubei Province, Wuhan Sports University, Wuhan, China

**Keywords:** Tai Chi, heel kick movement, statistical parametric mapping, stability, joint angle, center of mass

## Abstract

**Objective:**

The study aims to investigate the stability differences in the heel kick movement between Tai Chi athletes of different skill levels and explore the impact of Tai Chi training on postural control and stability.

**Methods:**

The study recruited 16 Tai Chi athletes, who were divided into two groups based on their skill level: the Elite Group (EG, 8 athletes) and the Sub-Elite Group (SG, 8 athletes). While performing the heel kick movement, the athletes’ kinematic data were captured using a Vicon motion capture system. The study compared the differences between the two groups by analyzing the changes in hip, knee, and ankle joint angles, and by using the SPM method. Additionally, the inter-group differences in center of mass (CoM) sway volume were also compared.

**Results:**

Significant differences in joint angles were observed, with EG showing greater stability in knee and ankle external rotation. During the knee-lifting phase, the CoM sway volume of the EG was significantly greater than that of the SG (*p* = 0.046), indicating larger postural adjustments in EG during this stage. No significant differences were found in the extension and recovery phases (*p* > 0.05).

**Conclusion:**

EG demonstrate superior joint stability and more efficient postural control strategies during the heel kick, particularly in the preparation phase. These findings highlight the role of advanced neuromuscular coordination in enhancing movement stability, providing biomechanical evidence to inform high-performance Tai Chi training.

## Introduction

The heel kick in Tai Chi is a representative and technically demanding movement that requires athletes to possess strong lower limb strength, joint flexibility, and the ability to maintain postural balance and stability during leg lifting and rapid kicking (Hosseini et al., 2018). The key to successfully executing this movement lies in whole-body coordination, particularly in achieving smooth execution and precise force control during rapid center of mass (CoM) transitions (Wehner et al., 2021). Postural stability is a key determinant of motor performance, particularly in dynamic movements like the heel kick (Wehner et al., 2021).

Tai Chi training has been associated with improvements in postural control and proprioception, especially in older adults and individuals with movement impairments (Li et al., 2001; Huang and Liu, 2015). These benefits are believed to result from the controlled, weight-shifting motions and slow, multi-planar movements characteristic of Tai Chi practice, which may support improved coordination and body awareness in dynamic scenarios. Some studies suggest that Tai Chi practitioners exhibit reduced postural sway and enhanced stability responses when exposed to challenging conditions or external perturbations, which may reflect improved CoM regulation and neuromuscular coordination (Sever et al., 2021). Additionally, Tai Chi offers notable advantages in improving gait stability and enhancing lower limb strength, which in turn reduces fall risks and improves dynamic balance. These benefits are particularly valuable for populations such as older adults and individuals with Parkinson’s disease (Chen et al., 2017; Chen et al., 2023; Cruz-Díaz et al., 2020).

In competitive sports, postural stability is equally critical for athletic performance. Dynamic balance and control capabilities are essential for all athletes, and these attributes become even more critical at elite levels, where executing complex movements with precision and consistency is crucial (Yue et al., 2023; Paillard, 2023). While previous studies have highlighted the unique role of Tai Chi in improving postural control, there remains a lack of systematic investigation into the differences in dynamic performance during the heel kick movement among athletes of varying skill levels. Understanding these differences can provide insights into the specific effects of training on postural control and offer scientific guidance for developing training strategies tailored to athletes with different technical proficiencies.

In sports, postural control is a core ability for maintaining body balance, performing complex movements, and preventing injuries. Postural control not only serves as the foundation for ensuring stability in both static and dynamic states but also plays a decisive role during movement, especially under rapid motion or rapidly changing conditions. Postural control works by continuously adjusting the coordination of different parts of the body, ensuring that athletes can respond to external disturbances, maintain balance, and perform movements accurately. Research has shown that good postural control significantly improves an athlete’s stability during movement, particularly in high-intensity sports (Horak, 2006). For example, Emilio et al. pointed out that postural control is crucial in preventing falls, especially among the elderly, where improved postural control can significantly reduce the risk of falls (Emilio et al., 2014). The association of flexibility, balance, and lumbar strength with balance ability: risk of falls in older adults.

Lower limb strength, flexibility, and coordination are key factors influencing postural control. Granacher et al. also emphasized the role of lower limb muscle strength in postural control, noting that good lower limb strength can help older adults maintain balance when facing sudden changes (Granacher et al., 2008). Especially for older adults or those with declining physical function, postural control training can significantly improve their balance in daily life and reduce the incidence of falls (Shumway-Cook and Woollacott, 2007). Shumway-Cook and Woollacott stated that postural control training, by enhancing lower limb strength and coordination, can help older adults improve their balance and reduce the risk of falls (Shumway-Cook and Woollacott, 2007). Postural control training typically works by strengthening lower limb muscles, improving muscle control, and enhancing body responsiveness. These training methods are crucial for enhancing an athlete’s balance performance in unstable conditions. In conclusion, understanding and training postural control is essential for improving athletic performance and preventing injuries. By using diversified training methods, especially lower limb strength and coordination training, athletes can effectively enhance their postural control, thereby improving their performance and stability in sports.

Statistical Parametric Mapping (SPM) is a statistical method designed for hypothesis testing in stochastic continuous data and is widely applied in the analysis of one-dimensional continuous variables in sports biomechanics research (Mei et al., 2021; Zhou et al., 2022; Kobayashi et al., 2022; Elad and Abramowitch, 2015). SPM not only enables comprehensive and objective analysis of one-dimensional multivariate vector field data, addressing biases in statistical testing of continuous data, but also corrects significance levels using random field theory to reduce the probability of Type I errors (Pataky et al., 2013; Wei and Wang, 2023). In the temporal analysis of lower limb biomechanics, SPM demonstrates high sensitivity to temporal inconsistencies, providing researchers with a tool to identify regions or time intervals where differences are most likely to be critical in movement execution. However, it is important to note that SPM cannot directly indicate where the differences are significant, but it can highlight areas of the data where differences are most likely to occur. This sensitivity makes SPM an effective method for detecting critical points in movement execution, which may be missed by traditional biomechanical methods. For example, Honert and Pataky emphasized the importance of timing in gait events and demonstrated how SPM can be used to identify key time points that influence whole trajectory analyses in lower limb biomechanics (Honert and Pataky, 2021).

This study aims to apply the one-dimensional multivariate vector field analysis method of SPM to examine the differences in joint angle characteristics and CoM sway volume among Tai Chi athletes of different skill levels during the heel kick movement. Through *post hoc* testing, we will identify specific time intervals contributing to these differences and further explore lower limb joint stability. From a biomechanical perspective, we will analyze the relationship between joint angle characteristics and postural control abilities among athletes with different technical levels. We hypothesize that: (1) The Elite Group will exhibit significantly different joint angle characteristics compared to Sub-Elite Group throughout the various phases of the heel kick movement. (2) The Elite Group will demonstrate more stable joint angle characteristics and a smaller CoM sway volume.

## Participants and methods

### Participants

The required sample size was estimated using G*Power 3.1.9.2 software, with a power analysis based on a large effect size (d = 0.8). To achieve a statistical power of 80% at a significance level of α = 0.05, a minimum of 16 participants was require (Penn et al., 2019; Wayne et al., 2014; Li et al., 2024). A total of 16 Tai Chi athletes were recruited for this study and categorized into two groups based on their skill level: The Elite Group (EG) (n = 8) consisted of athletes holding the title of National Master Athlete, awarded by the General Administration of Sport of China. These athletes had achieved podium placements in national-level competitions. and the Sub-Elite Group (SG) (n = 8) comprised athletes who held First-level Athlete Certificates, with experience in provincial- or municipal-level competitions but who had not yet reached elite status. The demographic characteristics of the participants are as follows: EG: Age (20.00 ± 3.83 years), height (173.00 ± 4.55 cm), weight (66.17 ± 7.24 kg). SG: Age (17.67 ± 1.70 years), height (173.17 ± 6.04 cm), weight (60.50 ± 3.25 kg). All participants were in good physical condition, with no history of injuries or medical issues in the past year. Before the experiment, they were provided with a detailed explanation of the study’s objectives, methods, and testing procedures. Each participant gave written informed consent before participating. This study was approved by the Medical Ethics Committee of Wuhan Sports University (Approval No. 2024048) and was conducted in compliance with the Declaration of Helsinki.

### Research methods

**FIGURE 1:**
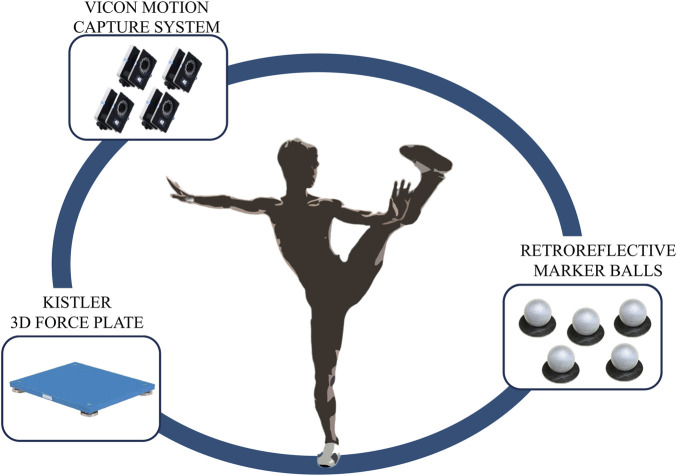
Experimental test and equipment diagram.

The experiment was conducted at the Key Laboratory of Sports Engineering of the General Administration of Sport of China, Wuhan Sports University. Motion capture data of the Tai Chi heel kick movement was collected using nine infrared high-speed cameras (T40, VICON, United Kingdom, sampling at 200 Hz). Ground reaction forces were collected using four KISTLER 3D force plates (9260AA6, Switzerland; 600 × 400 mm, 1,000 Hz). The experimental setup and equipment are shown in Figure 1. A full-body biomechanical model was built using Visual3D software (v6.01, C-Motion Inc., United States), based on the standard Plug-in Gait framework, and a total of 38 reflective markers were applied according to a standardized protocol.

Prior to testing, participants were informed about the experimental procedure and key precautions. Their basic demographic information was recorded, and key anthropometric measurements—including torso, upper arm, forearm, thigh, shank, and foot lengths—were taken. A total of 38 markers were securely attached to the participants’ skin following the predefined placement scheme. To minimize errors, participants were required to wear laboratory-standard compression garments, and all marker placements were consistently performed by the same experimenter.

Before formal data collection, a static trial was recorded to establish the baseline posture. Participants then performed a thorough warm-up before adjusting to the required stance. The formal motion capture session was recorded on video throughout. The experimenter guided participants to stand within the motion capture area, positioning both feet on two separate force plates. Following verbal instructions from the test administrator, participants performed the Tai Chi heel kick movement. Each participant completed six trials, with movements evaluated by an on-site Tai Chi coach to ensure compliance with technical standards. During the Tai Chi heel kick movement, all participants used their right leg as the support leg to ensure consistency and comparability of the movements.

### Data processing

After gap-filling and trajectory reconstruction in Vicon software, the motion capture data were exported as C3D files and imported into Visual 3D for full-body modeling and kinematic analysis. The kinematic data were smoothed using a low-pass Butterworth filter with a cutoff frequency set at 10 Hz (Jia et al., 2024; Liu et al., 2023). The joint coordinate system and joint angle definitions followed the default settings in Visual 3D, where the JCS was defined relative to the participant’s body and comprised three anatomical axes: the coronal axis (X-axis) for Flexion/Extension, the sagittal axis (Y-axis), representing Adduction and Abduction, which refer to movement toward and away from the body’s midline, respectively; and the vertical axis (Z-axis), representing Internal Rotation and External Rotation. In this study, joint angles are calculated by transforming one lower limb segment (such as the thigh) relative to another lower limb segment (such as the shank), using the local coordinate system of the shank as the reference frame. In this method, the position and orientation of the thigh (segment A) are described relative to the coordinate system of the shank (segment B). The angle value is obtained through this transformation relationship, which is commonly used to describe joint flexion or rotational movements. The center of mass (CoM) height is defined as a three-dimensional quantity relative to the laboratory coordinate system. Specifically, the position of the CoM involves changes along the three axes (x-axis, y-axis, and z-axis) in the laboratory coordinate system. In this coordinate system, the three-dimensional position of the CoM can be accurately described as a three-dimensional vector, where the z-axis component represents the vertical change in the CoM, while the x-axis and y-axis components reflect the position on the horizontal plane.

### Selection of evaluation metrics

This study primarily focuses on the stability of the supporting leg. Therefore, the hip, knee, and ankle joint angles of the right leg were selected for analysis. To evaluate postural stability, the center of mass (CoM) sway volume was used as a key metric, quantifying the degree of instability and sway amplitude of the CoM in different directions during motion (Xing et al., 2023). The calculation of CoM sway volume is based on the 95% confidence ellipsoid volume method proposed by Kutilek et al. (2015), with the specific formula as follows:

$$a=\sqrt{\lambda_{1}\cdot \chi_{\left(0.95,3\right)}^{2}}$$ (1)

$$b=\sqrt{\lambda_{2}\cdot \;\cdot \;\chi_{\left(0.95,3\right)}^{2}}$$ (2)

$$c=\sqrt{\lambda_{3}\cdot \;\cdot \;\chi_{\left(0.95,3\right)}^{2}}$$ (3)

$$V=\frac{4}{3}\pi abc=\frac{4}{3}{\cdot \left(\chi_{\left(0.95,3\right)}^{2}\right)}^{3/2}\cdot \sqrt{\lambda_{1}\lambda_{2}\lambda_{3}}$$ (4)

Here, V represents the sway volume of the center of mass; a , b , c are the semi-axis lengths of the confidence ellipsoid along the three principal directions. These axes are determined by the eigenvalues $\lambda_{1}$ , $\lambda_{2}$ , $\lambda_{3}$ of the covariance matrix, which represent the variance in three orthogonal directions, corresponding to the amplitude of marker oscillation in each direction. $\cdot \chi_{\left(0.95,3\right)}^{2}$ denotes the chi-square critical value at the 95% confidence level with 3 degrees of freedom.

### Phases of the heel kick movement

**FIGURE 2:**
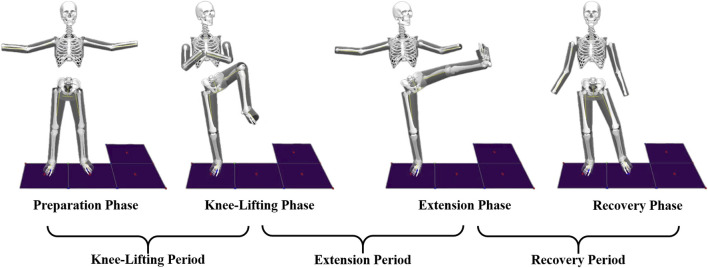
Schematic representation of the heel kick movement phases.

The segmentation of the heel kick movement was based on key kinematic and kinetic events commonly adopted in Tai Chi biomechanics research (Huang, 2024; Huang et al., 2024). The movement was divided into four primary phases: the Preparation phase (T1), marked by toe-off of the left (kicking) foot; the Knee-lifting phase (T2), ending when the left knee reaches maximum flexion; the Extension phase (T3), ending at maximum extension of the kicking leg; and the Recovery phase (T4), ending when the left foot contacts the ground again. For analytical purposes, the motion was further grouped into three functional periods: the Knee-lifting phase (T1–T2), beginning when the ground reaction force under the supporting leg starts to decrease and ending at peak knee flexion; the Extension phase (T2–T3), from maximum flexion to maximum extension of the kicking leg; and the Recovery phase (T3–T4), from peak extension to ground contact, identified by the return of force plate signals to baseline. A schematic representation of these phases is shown in Figure 2.

### Statistical methods

All statistical analyses were conducted using MATLAB R2020a. The significance level (α) was set at 0.05 for all tests. Statistical Normality tests were first performed on all variables to determine the appropriate statistical approach. If the data followed a normal distribution, parametric tests were applied; otherwise, non-parametric tests were used to compare the one-dimensional multivariate vector field of joint angle variables in the supporting leg across the three movement phase, SPM1d/SnPM1d was employed to perform Hotelling’s T^2^ test for independent samples, assessing differences between Tai Chi athletes of different skill levels. If the Hotelling’s T^2^ test rejected the null hypothesis, a *post hoc* analysis was conducted using SPM1d/SnPM1d independent sample t-tests, identifying specific regions where significant differences in joint angle variables occurred. For the analysis of CoM sway volume, either an independent sample t-test or the Mann-Whitney U test was applied, depending on the results of the normality test. To control for the risk of Type I errors due to multiple comparisons, Benjamini–Hochberg correction was applied to adjust p-values appropriately.

## Results

### Joint angle characteristics

**FIGURE 3:**
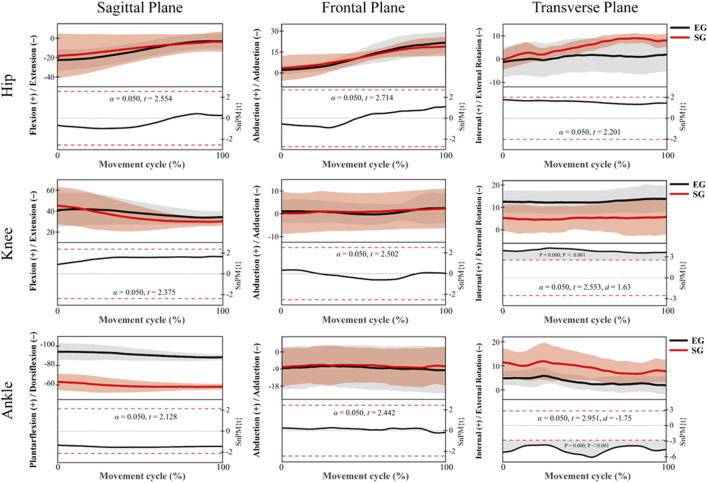
SPM{t} results comparing lower limb joint angles between EG (black) and SG (red) Tai Chi athletes during the knee-lifting phase. Joint angles are shown in three planes: sagittal (left), frontal (middle), and transverse (right) for the hip (top row), knee (middle row), and ankle (bottom row). Shaded regions represent standard deviations. α denotes the significance level (0.05); the t-value quantifies differences between groups or conditions, calculated based on sample means and standard errors; the effect size (Cohen’s d) indicates the magnitude of the difference between groups, with larger values reflecting more substantial differences, with the same convention applying to subsequent analyses.

The *post hoc* SPM{t} analysis results of lower limb joint angles during the knee-lifting phase are shown in Figure 3. For the hip joint, although inter-group differences were observed across the sagittal, coronal, and transverse planes, none reached the significance level (*p* > 0.05), indicating minimal differences between groups during this phase. For the knee joint, a significant region was identified in the transverse plane at approximately 100% of the knee-lifting cycle, where the EG exhibited significantly greater internal rotation than the SG (*p* = 0.000, Cohen’s d = 1.63). No significant differences were found in the sagittal or coronal planes. For the ankle joint, a significant region in the transverse plane was also observed at approximately 100% of the knee-lifting cycle, with the EG showing significantly greater external rotation than the SG (*p* = 0.000, Cohen’s d = 1.75), indicating a large effect size. No significant differences were found in the sagittal or coronal planes.

**FIGURE 4:**
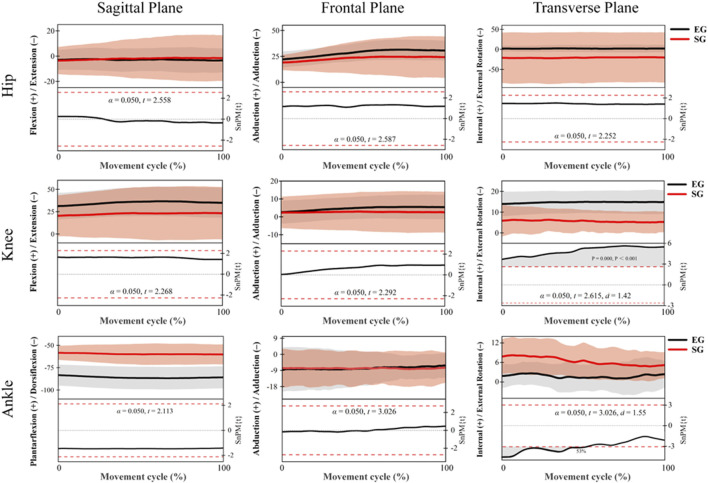
*Post hoc* SPM{t} analysis results of lower limb joint angles during the extension phase (EG, black. SG, red).

Figure 4 presents the *post hoc* SPM{t} analysis results for lower limb joint angles during the knee extension phase. For the hip joint, no significant differences were observed in any of the three anatomical planes (*p* > 0.05). Both groups exhibited similar flexion patterns in the sagittal plane, abduction in the frontal plane, and external rotation in the transverse plane, indicating comparable hip joint control strategies during this phase. For the knee joint, no significant differences were detected in the sagittal or frontal planes. However, a significant cluster was identified in the transverse plane, where the EG exhibited significantly greater internal rotation than the SG (*p* = 0.000, Cohen’s d = 1.42), indicating a large effect size. The significant region extended throughout the extension phase, suggesting notable group differences in axial control of the knee. For the ankle joint, no significant differences were found in the sagittal or frontal planes. In contrast, a significant cluster was observed in the transverse plane, where the SG showed significantly greater internal rotation than the EG across 0%–53% of the extension phase (*p* = 0.000, Cohen’s d = 1.55), also indicating a large effect size. These findings imply that the two groups employed different control strategies for rotational movement at the ankle joint.

**FIGURE 5:**
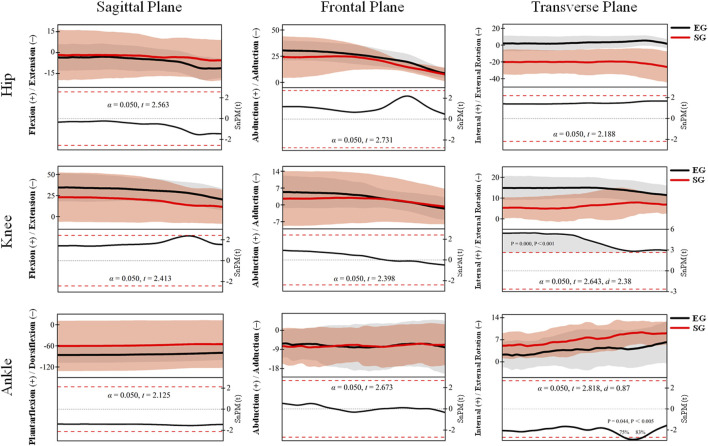
Joint angle trajectories and *post hoc* SPM{t} test results of the hip, knee, and ankle during the recovery phase (EG, black. SG, red).

Figure 5 illustrates the *post hoc* SPM{t} analysis results of lower limb joint angles during the recovery phase. No statistically significant differences (*p* > 0.05) were observed for the hip joint across all three anatomical planes (sagittal, frontal, and transverse). Both groups exhibited flexion in the sagittal plane, abduction in the frontal plane, and external rotation in the transverse plane, suggesting similar hip joint control strategies during this phase. For the knee joint, no significant differences were found in the sagittal and frontal planes. However, a significant region was detected in the transverse plane, where the EG exhibited significantly greater external rotation throughout the recovery phase compared to the SG (*p* = 0.000, Cohen’s d = 2.38), indicating a large effect size and notable differences in axial control of the knee between groups. In the ankle joint, no significant differences were observed in the sagittal and frontal planes. In contrast, a significant region was identified in the transverse plane between 75% and 83% of the recovery phase, where the SG demonstrated significantly greater external rotation than the EG (*p* = 0.050, Cohen’s d = 0.87), representing a medium-to-large effect size. This suggests distinct motor control strategies for axial ankle rotation between the two groups during the recovery phase.

### CoM sway volume

**FIGURE 6:**
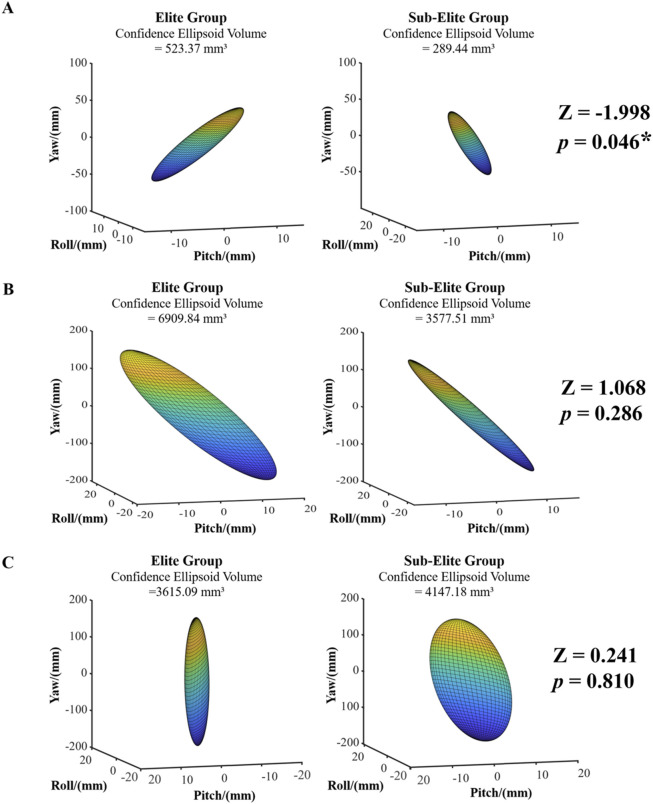
Center of mass sway volume and difference test results for athletes in different groups. **(A)** Knee-lifting phase. **(B)** Extension phase. **(C)** Recovery phase. A larger absolute Z-score indicates greater statistical significance between the two datasets, while the sign of the Z-score reflects the direction of the difference. Pitch angle corresponds to the x-axis, roll angle to the y-axis, and yaw angle to the z-axis. * denotes *P* < 0.05.

During the knee-lifting phase (Figure 6A), the CoM sway volume of the EG was significantly greater than that of the SG (523.37 vs. 289.44 mm^3^, *p* = 0.046). In the extension and recovery phases (Figures 6B,C), there was no significant difference between the groups (*p* > 0.05).

## Discussion

This study compared joint angle characteristics and CoM sway volume between Tai Chi athletes of different skill levels during the heel kick movement. The results revealed significant differences between the Elite Group and Sub-Elite Group in control strategies and movement execution along different axes, which is consistent with Hypothesis 1. However, although there was no statistically significant difference in CoM sway volume between the EG and SG during the extension and recovery phases (*p* > 0.05), the descriptive data indicated that EG had a larger value in the extension phase, whereas SG had a slightly larger value in the recovery phase, partially contradicting Hypothesis 2.

Our findings indicate that the EG demonstrated greater hip extension angles, suggesting superior control in hip flexion-extension. This superior control is evidenced by their ability to achieve greater joint range while maintaining postural stability, which reflects enhanced neuromuscular coordination and strength. Previous studies have shown that increased hip extension capacity is closely related to improved motor control strategies during dynamic balance tasks, as the hip plays a compensatory role in maintaining body stability, especially in the posterior kinetic chain during actions like kicking or landing (Winter, 1995). This result aligns with the study by Pohl et al. (2020), which highlights that as movement difficulty increases, the synergistic coordination of the hip, knee, and ankle joints plays a crucial role in maintaining balance. When performing movements on unstable surfaces, joint angle adjustments significantly enhance postural stability, which is closely linked to the precision of postural control developed through long-term training. Additionally, the EG exhibited greater hip abduction angles, indicating superior postural adjustment capabilities in maintaining CoM stability. This finding is consistent with (Zhu et al., 2021), who reported that Tai Chi movements can aid in dynamic balance maintenance and reduce joint load.

The differences in knee joint angles were primarily observed in flexion-extension and adduction-abduction angles, with the EG demonstrating greater flexibility and force output in dynamic knee joint control. In terms of flexion-extension angles, knee flexion plays a critical role in force transmission during the heel kick movement, as it facilitates energy transfer and shock absorption in lower limb kinetics (Pandy and Andriacchi, 2010). This study found that the EG exhibited a greater knee flexion range with lower fluctuation, indicating their ability to effectively utilize knee flexion for force transmission while maintaining postural stability. This finding is consistent with Markström et al., who reported that elite athletes demonstrate more stable knee joint flexibility and force output during lateral jumps, reflecting higher dynamic knee joint stability (Markström et al., 2019). In contrast, the SG exhibited a smaller knee flexion range with greater fluctuation, suggesting difficulty in maintaining stable force output during movement execution. This instability may be attributed to their limited dynamic control ability in the knee joint. Regarding adduction-abduction angle control, the EG demonstrated a complex adjustment pattern, transitioning from abduction to adduction and back to abduction, indicating their ability to adaptively adjust joint posture to accommodate complex movement demands. This finding aligns with Su et al., who reported that flexible knee joint posture adjustments in Tai Chi practice effectively reduce the risk of knee injuries and enhance movement precision (Su et al., 2022). Conversely, the SG exhibited a relatively fixed abduction pattern, lacking the ability to dynamically adjust knee joint posture, which suggests a limited capacity for postural adaptation during complex movements and difficulty in responding to rapid center of mass shifts.

The differences in ankle joint angles during the heel kick movement were primarily observed in flexion-extension, adduction-abduction, and internal-external rotation angles. As a key joint of the supporting leg, ankle postural control directly influences movement stability and force transmission efficiency. This study found that the EG exhibited a greater ankle extension angle during the supporting phase, indicating more stable ankle extension patterns and smoother movement execution. This finding is consistent with Su et al., who reported that incorrect postural control may increase joint stress, thereby compromising efficient force transmission and movement execution (Su et al., 2022). In contrast, the SG demonstrated a smaller ankle extension angle with greater fluctuation, indicating deficiencies in force transmission and postural control. Regarding adduction-abduction angle control, the EG exhibited a greater adduction angle, suggesting superior dynamic balance maintenance in the supporting leg. Adjustments in ankle adduction-abduction angles play a critical role in center of mass stability during complex movements. Langley et al. pointed out that ankle kinematics vary under different footwear designs, particularly in adduction-abduction angle control, which is essential for reducing the risk of sports injuries (Langley et al., 2019). In terms of internal-external rotation angle control, the EG demonstrated greater stability, particularly during the transition phase of the heel kick movement, effectively maintaining ankle joint stability and optimizing force transmission. Simpson et al. reported that ankle internal rotation control is crucial for stability in dynamic movements, as variations in internal rotation angles upon landing can influence movement smoothness and stability (Simpson et al., 2022). Conversely, while the SG also exhibited some degree of internal rotation, their greater fluctuation suggests a lack of precise ankle postural control during movement execution, leading to reduced movement stability.

These differences in CoM sway volume were associated with distinct balance control strategies adopted by Tai Chi athletes of varying skill levels. Specifically, a larger CoM sway volume may indicate greater neuromuscular engagement and exploratory postural adjustments, both of which are often observed in highly trained athletes striving to dynamically stabilize their posture under complex motor demands (Paillard, 2017; Donath et al., 2016). This interpretation differs from Hypothesis 2. This finding aligns with biomechanical research on Tai Chi Push Hands. Chang *et al.* found that practitioners maintain balance by controlling force rather than resisting it. Similarly, elite athletes in this study optimized force output and stability during the heel kick (Chang et al., 2014). Chen et al. further noted that experienced Tai Chi practitioners adjust posture to redirect external forces, reducing imbalance risk, which may explain the more stable kicking patterns observed in elite athletes (Chen et al., 2010). During the knee-lifting phase, the EG exhibited a significantly greater CoM sway volume than the SG, indicating that elite athletes made larger but controlled postural adjustments to stabilize the center of mass during the preparation phase. Porto et al. suggested that in the optimization of postural control, individuals must balance local optimization and global stability, particularly under visual and support constraints, where CoM adjustment strategies play a crucial role in overall stability maintenance (Porto et al., 2019). Although Tigrini et al. focused on Center of Pressure (COP) rather than Center of Mass (COM), their findings similarly suggest that smaller sway metrics are indicative of better postural control (Tigrini et al., 2021). In our task context, the EG’s larger yet regulated sway during the knee-lifting phase may facilitate subsequent movement execution, highlighting their advantage in long-term dynamic control training. In contrast, the SG showed a smaller CoM sway volume in this phase, suggesting more constrained adjustments and potentially less effective preparatory control.

There was no significant difference in CoM sway volume between the EG and SG during the extension and recovery phases (*p* > 0.05), which does not support Hypothesis 2. In the extension phase, EG showed a numerically greater CoM sway volume, suggesting a potentially more dynamic balance-control strategy. Holmes et al. found that in high-difficulty movements, allowing moderate CoM fluctuation can enhance force transmission efficiency, and this strategy is often associated with the training experience of elite-level athletes (Holmes et al., 2016). Conversely, the SG’s smaller value in the extension phase should be interpreted as a non-significant tendency rather than a confirmed group difference.

During the recovery phase, SG exhibited a numerically greater CoM sway volume than EG, although the difference was not statistically significant. This tendency may reflect different post-movement stabilization strategies: EG appeared to regain balance with smaller sway, indicating a more efficient stabilization process, whereas SG required slightly larger adjustments to restore equilibrium. Such phase-specific differences in CoM sway volume suggest that athletes at different skill levels adopt distinct approaches to balancing force regulation and postural control. Ilham et al. reported that muscle synergy-based measurement methods can enhance the stability of electromyographic (EMG) signals during movement, improving comparability across movement strategies and thereby supporting more effective postural control and force output (Ilham et al., 2024). Similarly, Vando et al. found that elite athletes can rapidly re-establish postural stability after high-intensity actions, a capability closely linked to long-term postural adjustment training (Vando et al., 2021). Overall, the variations in CoM sway volume across the heel-kick phases underscore pronounced differences in balance-control and force-regulation strategies between skill levels. EG demonstrated more adaptable and efficient CoM adjustments, enabling a better balance between force output and stability maintenance, whereas SG showed limitations in fine-tuning CoM control.

Future studies will further investigate CoM control and force output strategies across different Tai Chi movement phases, incorporating surface electromyography techniques to explore the interaction between joint control and force production. Additionally, research will focus on enhancing dynamic balance and postural control in lower-level athletes by optimizing training methodologies, aiming to reduce the technical gap between skill levels and improve overall athletic performance.

## Conclusion

This study shows that, compared with sub-elite athletes, elite Tai Chi athletes exhibit a significantly greater CoM sway volume during the knee-lifting phase, reflecting superior dynamic postural adjustment capacity and neuromuscular coordination in the preparation stage. Although no significant differences were found during the extension and recovery phases, the two groups displayed distinct balance control strategies, highlighting the importance of advanced Tai Chi training in optimizing phase-specific postural adjustments and stability.

## Data Availability

The raw data supporting the conclusions of this article will be made available by the authors, without undue reservation.
