# Supplementary figures and images for: Study on stability differences in heel kick movements of Tai Chi athletes based on statistical parametric mapping
Source: Front Physiol. 2025 Aug 29;16:1629653. doi: 10.3389/fphys.2025.1629653 (PMC12425959; doi:10.3389/fphys.2025.1629653)

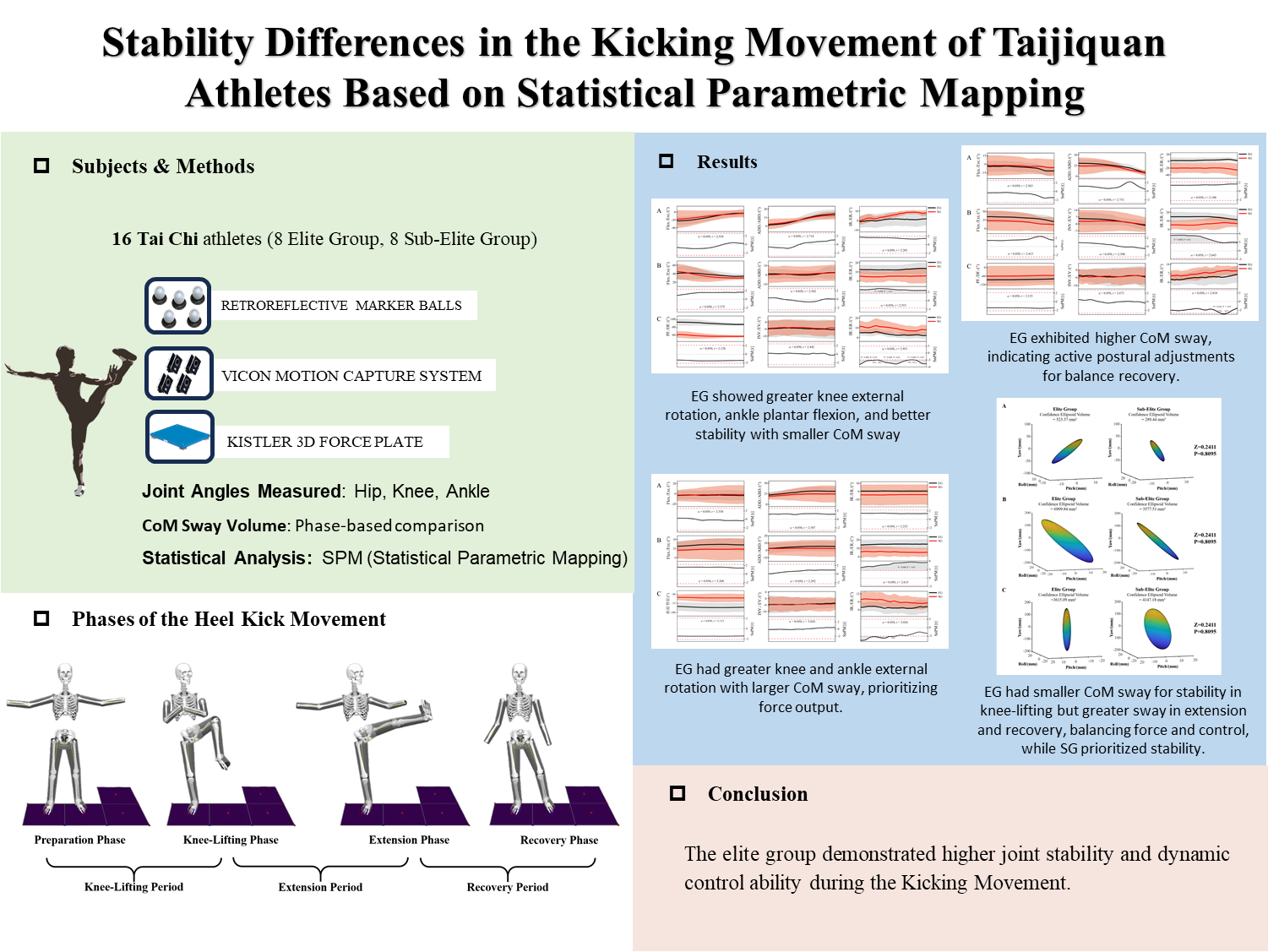

Supplement: Supplementary file 1 [file Image1.tif]
